# On the Successful Use of Very Large Doses of Opium in Tetanus

**Published:** 1817-05

**Authors:** John Taunton

**Affiliations:** Member of the College of Surgeons, and Lecturer in Anatomy and Physiology.


*For the London Medical and Physical Journal.*

### *On the successful Use of very large Doses of Opium in Tetanus;* by JOHN TAUNTON, Esq. Member of the College of Surgeons, and Lecturer in Anatomy and Physiology.

IN the treatment of Tetanus, it has been my practice to give opium in larger doses than it has been usually given, generally in a liquid form, often upwards of two ounces of the best tincture within twenty-four hours. The safety and success of this practice has been such as to induce the recommendation of giving very large doses of opium in this most formidable malady.

Mr. Reid, author of the subjoined letter, who attended my lectures in 1809, previously to his being appointed a surgeon in the royal navy, has given this medicine in still larger doses than I have ever done, and with much more success. I believe he has not lost one patient. It should be remarked, that, although tetanus is much more frequent in warm climates, it is not so generally fatal as it is in this.

In June, 1815, this practice was completely successful in a well-marked case, which, from the violence of the symptoms, threatened a fatal termination. A boy, about twelve years of age, was bitten on the hand and fingers; the parts were much lacerated and punctured. At the time I first saw him, the head was bent forwards on the chest; the upper and lower part of the trunk were drawn forcibly back; the jaw was completely locked; the spasmodic action of the muscles produced violent pain. Directions were given to foment and poultice the hand and fingers; and the following medicine was prescribed:

℞. Liquoris Ammoniæ Acetatis ℥iij.—Tincturæ Opii ℥j. Misce, cochleare unum magnum 2da quâque horâ sumendum.

The symptoms began to yield on the second, and were nearly removed on the sixth, day. A copious perspiration succeeded the exhibition of the medicine, and seemed to be productive of good. In many cases, however, we find the most profuse perspiration, without the least alleviation of symptoms.—The wounds on the hand were several weeks before they were completely healed.

In those instances where the practice has not succeeded, it has appeared to me that either the opium has been given *too sparingly*, or the dose diminished *too soon*; for, when the dose has been lessened after only a partial remission of symptoms, the disease has returned with increased violence, and proved fatal.

In

In the Edinburgh Medical and Surgical Journal for July 1815, page 306, a successful case is related by Dr. Reid of Kilmarnoch, treated by opium, wine, and mercury; on the last remedy the author lays great stress.

Dr. Morrison, late of Demarara, in a letter communicated to Dr. Hancock, says, "I have seen, since I came to this country (in the year 1808), about thirty-two cases of tetanus, of which about seven got well. I gave one patient *eight grains of opium every two hours.* He recovered."

---

*His Majesty's Ship Strombolo, off the*

MY DEAR SIR, *Ebro, Jan. 2, 1814.*

During the periods I had the honour of attending your lectures, I was struck with a practice you recommended, of administering large doses of opium in spasmodic, but more particularly in well-marked tetanic, complaints. A short time after this, I had occasion to attend three patients labouring under that dreadful malady locked-jaw, to all of whom opium was given in larger doses than, I believe, the generality of practitioners have yet ventured to give. I think I mentioned the particulars and happy result of this treatment, when I last had the pleasure of seeing you.*

A very interesting case occurred to me lately, which I shall take the liberty to annex, in the undigested state of my notes taken at the patient's bed side.

I think, from analogy, were a case of hydrophobia to come under my care, I should be inclined to try the effect of opium, pushed as far as might be consistent with propriety. Permit me to return my sincerest thanks for the advantages and disinterested friendship I experienced from you while I was your pupil. I humbly beg that you will solicit the attention of the gentlemen who attend your anatomical theatre to the following subject; and more especially of those who have it in contemplation to visit hot countries. Sincerely hoping that this practice may prove as satisfactory and successful in the hands of others as it has done in mine, I remain, my dear Sir, with inexpressible sentiments of respect and esteem,

Your obliged and humble Servant,

THOMAS READ.

*To John Taunton, Esq. Hatton Garden.*

---

* In 1813, Mr. Reid related to me three cases of tetanus that he had treated successfully by opium. He gave first ten grains of the best solid opium every hour, till the symptoms had yielded in a considerable degree; then he lessened the dose gradually, but in each of the cases he gave upwards of two ounces of the best solid opium within four days.—J. T.

Nov.

Nov. 26, 1813.—I was requested to visit a Spanish peasant, who had received a very extensive contused wound on the right heel. Previously to this, he had been attended twenty-five days by a Spanish surgeon, who very inhumanly declined further attendance, on finding that his patient was no longer able to pay the wonted fee, a shilling per visit. When I first saw him, the wound was nearly filled with healthy granulations, but his general health seemed much impaired. He complained of excruciating pains in his legs, thighs, and belly, with stiffness and rigidity in the muscles of the neck; deglutition was performed with extreme difficulty. I recommended the warm bath; but, as our ship lay at a considerable distance, I had no opportunity that night of sending him any medicine. I visited him the next day, at eleven o'clock, and found all the symptoms greatly aggravated. He had general and violent spasms; his teeth were so closely shut that a knife could not be introduced between them; his pulse was 93, and scarcely perceptible. In the space of twenty-three minutes a remission ensued, of which I took advantage, and administered the following bolus:

℞. Extr. Opii gr. lxiv.—Camphor gr. x. Misce.

I left an ounce of genuine extract of opium formed into five grain pills, with directions to give two every hour, until I called again.

28th.—Owing to the bad state of the weather, I was prevented from going on shore.

29th (twelve o'clock).—He took the last dose of opium a little before I arrived. Has had no return of spasms since five o'clock the preceding evening, and has had six hours sound sleep. Pulse 82, soft, and full. Wound looks worse. I directed it to be dressed with Ung. Resin. Flav.

℞. Submuriat. Hydrar. ℈j.—Pulv. Jalap. gr. x. Misce. Sumat statim.—Rep. Pil. ex Opio 2da quâque horâ.

30th.—Has had two dark-coloured and fœtid stools, and three paroxysms, since yesterday, but less violent than before. The opium was now given in ten-grain doses every hour, as formerly.

Dec. 1st.—Appearances are greatly improved. Has had no return of the spasms. A nutritive regimen was provided, by a subscription raised amongst the English.—Rep. Pil. j. omni hora.

2d.—He is free from pain and spasm; his appetite improving; the wound cicatrizing. Let the opium be omitted.

3d.—I found him sitting up in bed quite cheerful, with no other complaint than debility. Wound nearly healed.

4th;

4th.—Bowels torpid. Capt. Ol. Ricin. ℥iſs. Complains of sleepless nights. Capt. Opii Purif. gr. x. hora somni.

5th.—Has had a good night; bowels regular.

℞. Pulv. Cinchon. ℈j.—Vin. Rubi ℥iij. tertia quâque hora sumendus.

This plan was continued till the 27th, when he was perfectly well, and undertook a journey on foot to Valencia, a distance of nearly one hundred miles.

In this case, 534 grains of opium were taken in forty-eight hours, 120 the next twenty-four hours or third day, 240 the fourth, and 120 the fifth,—in all 1014 grains in 120 hours; yet neither delirium nor any unusual costiveness attended this extraordinary quantity. THOMAS REID.

